# In Silico Investigation of SNR and Dermis Sensitivity for Optimum Dual-Channel Near-Infrared Glucose Sensor Designs for Different Skin Colors

**DOI:** 10.3390/bios12100805

**Published:** 2022-09-29

**Authors:** Murad Althobaiti

**Affiliations:** Biomedical Engineering Department, College of Engineering, Imam Abdulrahman Bin Faisal University, Dammam 31441, Saudi Arabia; mmalthobaiti@iau.edu.sa

**Keywords:** bioinstrumentation, dual-channel, glucose, near-infrared, NIR technology, sensors

## Abstract

Diabetes is a serious health condition that requires patients to regularly monitor their blood glucose level, making the development of practical, compact, and non-invasive techniques essential. Optical glucose sensors—and, specifically, NIR sensors—have the advantages of being non-invasive, compact, inexpensive, and user-friendly devices. However, these sensors have low accuracy and are yet to be adopted by healthcare providers. In our previous work, we introduced a non-invasive dual-channel technique for NIR sensors, in which a long channel is utilized to measure the glucose level in the inner skin (dermis) layer, while a short channel is used to measure the noise signal of the superficial skin (epidermis) layer. In this work, we investigated the use of dual-NIR channels for patients with different skin colors (i.e., having different melanin concentrations). We also adopted a Monte Carlo simulation model that takes into consideration the differences between different skin layers, in terms of blood content, water content, melanin concentration in the epidermis layer, and skin optical proprieties. On the basis of the signal-to-noise ratio, as well as the sensitivities of both the epidermis and dermis layers, we suggest the selection of wavelengths and source-to-detector separation for optimal NIR channels under different skin melanin concentrations. This work facilitates the improved design of a compact and non-invasive NIR glucose sensor that can be utilized by patients with different skin colors.

## 1. Introduction

Diabetes is a long-lasting health condition that impacts the process of turning food into energy in the human body, commonly known as metabolism. Over time, diabetes can lead to serious health issues, such as heart disease, kidney disease, and vision loss. Therefore, the regular monitoring of blood glucose levels is vital for diabetic patients. Over the past few years, scientists and engineers have developed practical invasive and non-invasive techniques that allow patients to regularly monitor their blood glucose level. Invasive electrochemical sensors are considered to be the gold standard for measuring blood glucose [1]. The review article [2] comprehensively investigated recent advancements in non-invasive blood glucose sensors that are optical, electrochemical, and microwave-based sensors. Non-invasive microwave blood glucose sensors have attracted the attention of many researchers due to their high skin penetration depth and low cost. Nevertheless, the sensitivity of microwave-based sensors still needs to be improved to be clinically accepted [2]. Non-invasive electrochemical reaction techniques are currently available, but their accuracy and lifetime are limited [3,4,5]. Moreover, continuous glucose monitoring (CGM) electrochemical devices are currently in use. CGM devices are minimally invasive techniques based on an implanted needle [6,7]. The article [8] identified 34 non-invasive and 31 minimally invasive glucose monitoring products, and it reviewed their regulatory, technological, and consumer features.

Optical glucose measurement devices are emerging and promising techniques. These techniques have the advantages of being non-invasive, compact, and user-friendly devices [9,10]. Photoacoustic spectroscopy [11], optical coherence tomography [12,13], Raman spectroscopy [14], and near-infrared (NIR) technology are all non-invasive optical techniques that have been investigated for the measurement of glucose. One study [10] reviewed the recent developments of different optical techniques, and their features and limitations were also highlighted. NIR techniques are the most-used and -studied optical techniques, due to their compactness and low cost. They contain three main parts—an NIR source, a tissue sample, and a photodiode—to detect the scattered or attenuated transmitted NIR light. NIR spectroscopy has been utilized in many medical applications, such as neuroimaging [15,16], the detection of breast cancer [17,18,19], and for blood glucose measurements and monitoring [20,21,22].

There have been great efforts in the scientific community to tackle the complexity of skin optical measurements. Notably, the article [23] studied the effect of changing the glucose concentration on light transport using a Monte Carlo simulation model. It is evident that, with a single wavelength approach, there is a potential challenge to measure glucose concentration due to the optical complexity of the skin. Another study [24] proposed an optical probe model with two concentric rings to measure the reflected optical signals at two different positions. This allowed estimating the variations in skin optical properties by variations in the blood glucose level. The authors of [25] proposed a technique using Monte Carlo simulation to reduce glucose prediction errors produced by temperature and scattering variations. The authors found that small changes in the temperature or volume fraction of the scattering particle would lead to large glucose prediction errors.

In a previous study [26], we introduced an optimized NIR sensor with two channels for blood glucose measurements. The long channel is utilized to measure the glucose level in the inner skin (dermis) layer. This measured signal carries important information regarding the glucose content. The short channel is used to estimate the interference noise arising from the superficial skin (epidermis) layer. Thus, the long channel signal can be used to determine the glucose content in the dermis layer, and the short channel signal can then be eliminated from the long channel signal. The dual-channel NIR sensor approach uses two sources with different wavelengths. The two wavelengths of the two sources and source–detector separation (SDS) were determined on the basis of a Monte Carlo simulation (MCS) model. The module was specifically investigated for the NIR wavelength range between 1200 and 1900 nm. However, this model does not consider the detailed anatomical features of skin layers, such as blood, water, and melanin concentrations. These parameters are important to consider specifically when investigating the diagnostic window of the NIR spectrum, which ranges between 450 and 1000 nm [16,27,28].

In this manuscript, we systematically studied the effect of the diagnostic window of the NIR wavelength spectrum, the effect of different skin colors (i.e., different skin melanin concentrations), and the source-to-detector separation (SDS) of these wavelength ranges on the optimal selection of the short and long NIR channels. In addition, an improved and more detailed skin model [29,30,31,32] was adopted for Monte Carlo simulation (MCS). This model takes into consideration the differences between different skin layers, in terms of the blood volume fraction, water volume fraction, melanin concentrations in the epidermis layer, and optical skin proprieties. The absorption of this NIR range (from 450 to 1050 nm) by the melanin of the epidermis layer and by different dermis layers differs. Therefore, we expect the optimal selections of the short and long NIR channels to be different for different wavelengths and for different skin colors.

## 2. Methods

### Monte Carlo Skin Model

**Figure 1:**
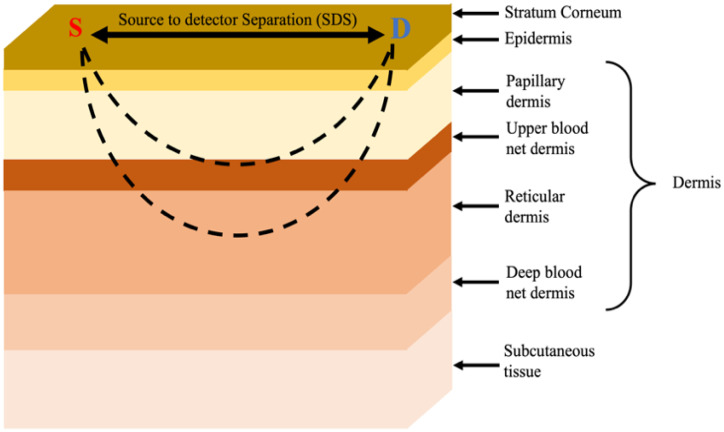
Schematic drawing of the skin model for Monte Carlo simulation. The light propagation distribution, having a “banana shape”, is illustrated by the dashed lines between the source (S) and the detector (D).

The Monte Carlo simulation (MCS) method was used in this study, which was described in [33,34]. The light source was modeled as a pencil beam light towards the z-direction. A detector with a radius of 2 mm is located at a distance from the source as shown in Figure 1. The photons detection replay mode described in the paper [35] was utilized. In brief, the method, initiated by launching millions of photons (here, 100 million) and the propagation of any launched photon in the skin layers, is calculated on the basis of the optical properties of the tissues.

The skin media are represented as a 3D volume, and each section in the volume is labeled to represent a specific layer of the skin. Therefore, the location of each voxel in the skin layers is pre-identified. At the reflection interface, the reflection coefficient is calculated based on Fresnel’s equation. The coefficient is then multiplied by the photon packet weight. For more details, the reader is referred to [33,34]. The history of each propagated photon is tracked with prior knowledge of the optical properties of different skin layers. In the MCS of the tissue, we considered the absorption coefficient (*μ_a_*), scattering coefficient (*μ_s_*), and refractive index (*n*) of each skin layer. These optical properties are all wavelength-dependent. As a photon travels deeper into the tissue, it loses its energy, which results in a low signal-to-noise ratio (SNR) at the detector side. Therefore, with a long SDS, one can measure deeper layers; however, this requires a highly sensitive detector to measure signals with a low SNR. On the other hand, with a short SDS, one can detect photons that are scattered from superficial layers with a good SNR. Therefore, the choice of both the operating source wavelength and the optimal SDS is critical in the design of both the long and short channels for NIR glucose sensors.

For this study, a skin model was built to mimic the propagation of light photons in the NIR diagnostic window, which ranges from 450 to 1050 nm, with an increment of 100 nm. It is also worth noting that, when choosing the operating source wavelengths, [36] was considered for spectral glucose absorptivity. The spectral range 450–1050 nm, known as the “diagnostic window”, has attracted the interest of researchers for many different diagnostic applications because water absorption is at its minimum [10,16]. This allows light to penetrate deeper into the tissue. In [36], the authors showed the wavelength-dependent absorptivity of glucose in an aqueous solution and a glassy state. This range is less sensitive for temperature changes on the absorptivity of glucose in comparison to longer wavelengths (>1200 nm).

The anatomical skin model consisted of seven layers, where the layers were optically inhomogeneous. The different skin layers are illustrated in Figure 1.

In this model, according to [29], the absorption coefficients for each dermis layer were calculated considering the differences of important anatomical parameters between different layers:

(1) $$\mu_{a}^{layer}\left(\lambda \right)=\left(1-S\right)\gamma V_{blood}\mu_{a}^{Hb}\left(\lambda \right)+S\gamma V_{blood}\mu_{a}^{HbO_{2}}\left(\lambda \right)\;+(1-\gamma V_{blood})\;V_{H_{2}O}\;\mu_{a}^{H_{2}O}\left(\lambda \right)+\left(1-\gamma V_{blood}\right)\;\left(1-V_{H_{2}O}\right)\mu_{a}^{other}\left(\lambda \right),$$

where $V_{blood}$ and $V_{H_{2}O}$ are the blood and water volume fractions, respectively; $\mu_{a}^{H_{2}O}$*,* $\mu_{a}^{Hb},$ and $\mu_{a}^{HbO_{2}}$ are the absorption coefficients for water, deoxyhemoglobin, and oxyhemoglobin, respectively; γ is calculated on the basis of the assumption that hemoglobin is only contained in the erythrocytes, which is zero for the stratum corneum and epidermis layers and 0.1 for the dermis layers [29]; $\;\mu_{a}^{other}$ is the calculated absorption coefficient for hemoglobin-free tissue, which can be estimated as follows [29,37]:

(2) $$\mu_{a}^{other}\left(\lambda \right)=7.84\times 10^{7}\times \lambda^{-3.25}.$$

According to [29,31], the absorption coefficients (*μ_a_*) for the stratum corneum and the epidermis layers are calculated as follows:

(3) $$\mu_{a}^{Stratum}\left(\lambda \right)=\left(0.1-8.3\times 10^{-4}\times \lambda \right)+0.125\times \mu_{a}^{other}\left(\lambda \right),$$

(4) $$\mu_{a}^{epidermis}\left(\lambda \right)=V_{mel}\;\mu_{a}^{mel}\left(\lambda \right)+V_{H_{2}O}\;\mu_{a}^{HbO_{2}}\left(\lambda \right)+\left(1-\left(V_{mel}+V_{H_{2}O}\;\right)\;\right)\;\mu_{a}^{other}\left(\lambda \right),$$

where $\mu_{a}^{mel}$ is the melanin absorption coefficient, estimated as

(5) $$\mu_{a}^{mel}\left(\lambda \right)=6.6\times 10^{10}\times \lambda^{-3.33}.$$

According to the values reported in [38,39] for melanosome volume concentrations ($V_{mel}$) in the epidermis layer for people having different skin colors, $V_{mel}$ ranges between 1% and 3% for light-skinned Caucasians, from 11% to 16% for Mediterranean people, and from 18% to 43% for darkly pigmented Africans. In this study, we used values of 2%, 10%, 20%, and 30% to study the effect of the melanin concentration on the optimal selection of the NIR channels. 

**Figure 2:**
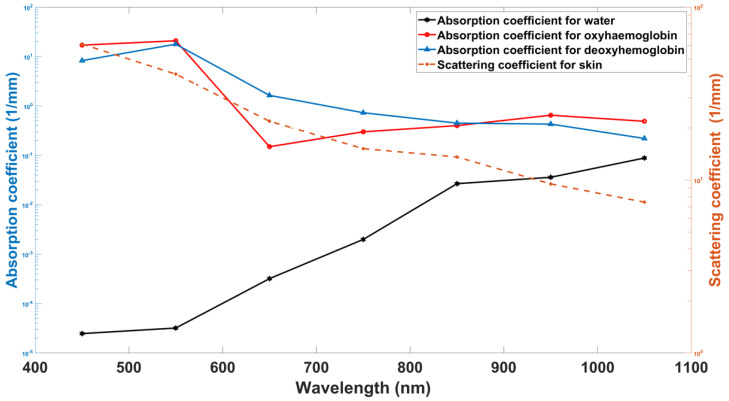
Other optical properties values in the utilized in MCS model: scattering coefficients *μ_s_* and absorption coefficients for water ($\mu_{a}^{H_{2}O}$), deoxyhemoglobin ($\mu_{a}^{Hb}$), and oxyhemoglobin ($\mu_{a}^{HbO_{2}}$) [27,31,38].

Table 1 summarizes the values utilized in Equation (1) for the estimation of the absorption coefficients of the various skin layers. The values of other optical properties utilized in this model, including the scattering coefficients *μ_s_* and the absorption coefficients for water ($\mu_{a}^{H_{2}O}$), deoxyhemoglobin ($\mu_{a}^{Hb}$), and oxyhemoglobin ($\mu_{a}^{HbO_{2}}$), are illustrated in Figure 2. The refractive index values used in this model are 1 for air and 1.4 for tissue [31].

To systematically assess the performance when changing the wavelength and the SDS in order to choose the optimal NIR channel for measuring glucose content, we previously introduced [26] three metrics. Briefly, the first metric is the epidermis sensitivity, which is the summation of the photon density function (PMDF) for all voxels in the epidermis layer over the summation of all photon density functions (PMDF) in the model:

(6) $$ES=100\times \frac{\sum_{Epidermis\;}\;\mathrm{PMDF}\;}{\sum_{total,\;}\;\mathrm{PMDF}\;}$$

The PMDF is computed by taking the voxelwise product of the fluence distribution of the source and the fluence distribution of the detector; details on the computation of the PMDF can be found in [40]. The second metric is the dermis sensitivity, which is calculated similarly to that for the epidermis, as follows:

(7) $$DS=100\times \frac{\sum_{Dermis\;}\;\mathrm{PMDF}\;}{\sum_{total,\;}\;\mathrm{PMDF}\;}\;$$

These metrics can provide an indication of how sensitive a particular NIR channel (with a specific wavelength and SDS) is to the epidermis and the dermis layers, respectively.

The third metric involves the calculation of the SNR for each NIR channel. As the SDS increases, light penetrates deeper into the tissue, i.e., the dermis layer, but the SNR decreases. Therefore, there is a tradeoff between a good SNR and a high depth of light penetration into the tissue. Thus, a balance between high dermis sensitivity and an acceptable SNR should be carefully considered. 

By running the MCS for multiple independent seeded simulations, one can calculate the mean (μ) and standard deviation (*σ*) at each voxel in the model. Thus, one can calculate the SNR (in decibels) as follows [26]:

(8) $$\mathrm{SNR}\left(SD\right)=20{log}_{10}\frac{µ\left(SD\right)}{\sigma \left(SD\right)}$$

The SNR is calculated for all MCS models, i.e., for all different wavelengths and all different ranges of the SDS. To calculate μ and σ, all the voxels in the model are considered. The calculation of the SNR was introduced and detailed [41]. As indicated above, the study was conducted considering various important parameters. First, the simulation was performed for the wavelength range from 450 to 1050 nm with an increment of 100 nm. For each wavelength, the simulation was completed for a range of source-to-detector separations (SDSs); specifically from 0.5 to 8 mm with a step size of 0.5 mm. In a previous study [26], we found that, by running multiple independently seeded MCSs (*N* = 15 to 30) for each wavelength and SDS, one could achieve an acceptable convergence when calculating the SNR. Here, *N* = 15 was sufficient for calculating the SNR for longer wavelengths, while *N* = 20 was sufficient for shorter wavelengths. For consistency, we adopted *N* = 20 for all MCS runs.

## 3. Results and Discussion

**Figure 3:**
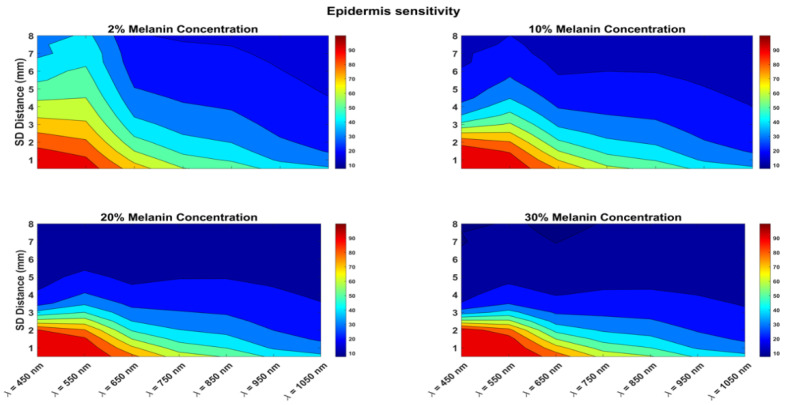
Epidermis sensitivity for various melanin concentrations and with the wavelength ranging between 450 and 1050 nm. Sensitivity also shown for SDS ranging from 0.5 to 8 mm.

The calculated sensitivity for the epidermis layer is shown in Figure 3. The epidermis sensitivity was calculated for different melanin concentrations, ranging from 2% (which represents light skin) to 30% (which is for dark skin). The figure also shows the effect of different wavelengths (450 to 1050 nm) and the effect of increasing the SDS from 0.5 to 8 mm. There was a clear reduction in epidermis sensitivity when increasing the melanin concentration from 2% to 10%, but the reduction in epidermis sensitivity was not strong when the melanin concentration was further increased from 20% to 30%. In darker skin, the epidermis layer was very sensitive to wavelengths from 450 to 650 nm for an SDS of up to 2.5 mm. For light skin (2% melanin concentration), the epidermis layer was very sensitive to the same wavelengths from 450 to 650 nm, and the SDS could be up to 4.5 mm.

**Figure 4:**
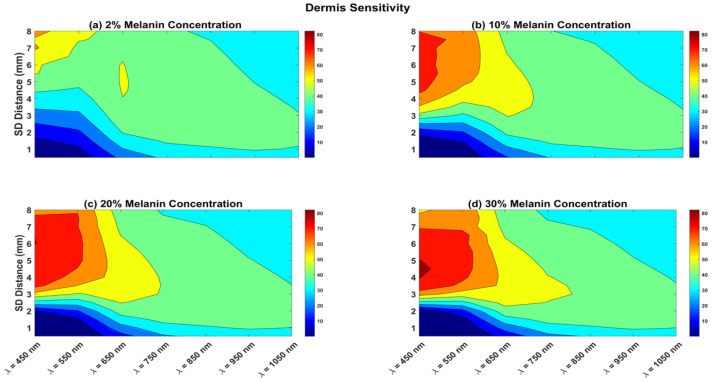
Dermis sensitivity for various melanin concentrations and with the wavelength ranging between 450 and 1050 nm. Sensitivity also shown for SDS ranging from 0.5 to 8 mm.

Similarly, the calculated sensitivity for the dermis layers is shown in Figure 4. For shorter wavelengths, dermis sensitivity increased as the melanin concentration increased. The SDS from 4 to 8 mm had high dermis sensitivity. However, for a 2% melanin concentration, dermis sensitivity was only 50% when the SDS was longer than 5.5 mm. For darker skin, the wavelengths of 650 and 750 nm had 50% dermis sensitivity when the SDS was longer than 3 mm. For all different melanin concentrations, wavelengths from 450 to 650 nm had the lowest dermis sensitivity (less than 20%) with the SDS less than 2.5 mm. For all wavelengths at all different melanin concentrations, for an SDS of 1.5 mm or less, dermis sensitivity was always less than 30%.

**Figure 5:**
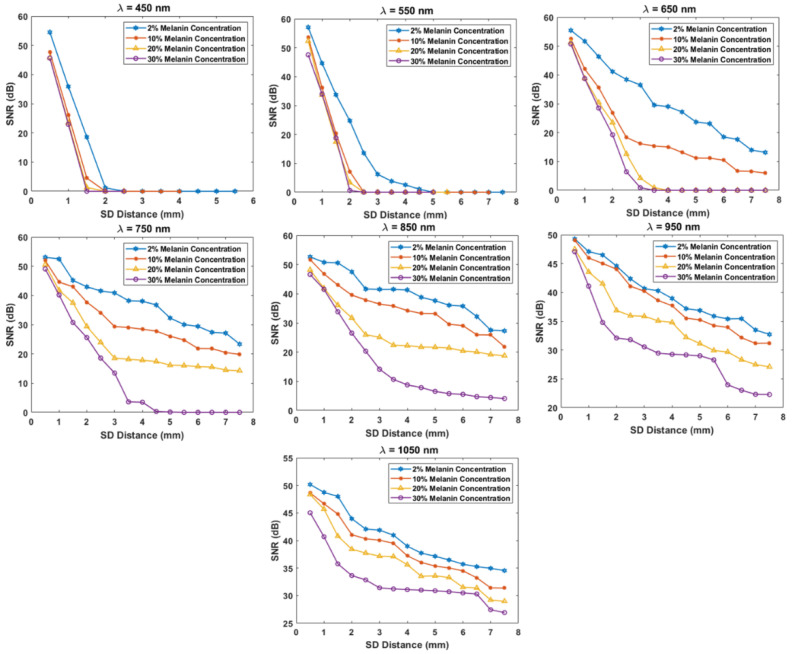
Calculated SNR for all different melanin concentrations, wavelengths, and SDSs.

Figure 5 shows the calculated SNR for all scenarios. As the melanin concentration increased, the SNR clearly deceased. For lighter skin (2% melanin concentration) and wavelengths between 650 and 1050 nm, the SNR was always above 15 dB. As the wavelength increased, the general trend of the SNR increased as well. For wavelengths of 450 and 550 nm, the SNR was almost zero for darker skin when the SDS was longer than 1.5 mm. The SNR was better for light skin at these two wavelengths, where the signal could be measured up to 1.5 mm at 450 nm and up to 4 mm at 550 nm.

At a 30% melanin concentration, one needs to go longer than the 750 nm wavelength to have a good SNR with a longer SDS. More specifically, the 850 nm wavelength had an SNR of 5–10 dB with the SDS ranging from 4–7.5 mm. For the same SDS range, a wavelength of 950 nm had a better SNR, of 20–30 dB. Similarly, the SNR was greatly improved for the same SDS range when increasing the wavelength to 1050 nm, to about 25–32 dB. 

As the SDS increased, the general trend of the SNR decreased. In comparison to light skin, the SNR in darker skin decreased at a higher rate when the SDS increased. For an SDS of 0.5 to 1 mm, the SNR range was always above 20 dB for all different melanin concentrations and wavelengths. 

In the design of dual-channel NIR glucose sensors, epidermis and dermis sensitivity, as well as SNR factors should be considered. From a practical perspective, the channels should have enough source-to-detector separation such that the sensor is easy to design and build. The aim of the short channel is to suppress the noise arising from the superficial epidermis layer. In contrast, the long channel is employed to measure the glucose content confined in the blood-containing inner dermis layer. 

For light skin (2% melanin concentration), wavelengths of 450 and 550 nm at an SDS of up to 2.5 mm showed the highest epidermis sensitivity (Figure 3). However, looking at the SNRs of the two wavelengths at the same SDS range, it is clear that a wavelength of 550 nm at a 2.5 mm SDS is a better choice for the short channel. For the long channel, we looked for the highest dermis sensitivity (Figure 4), which was at the long SDS, and the SNR was only good for longer wavelengths. Therefore, the optimal long channel uses the wavelength of 650 nm with an SDS between 4 and 6 mm. 

For skin with a 10% melanin concentration, epidermis sensitivity was 80% at 550 nm and 70% at 650 nm at a 2 mm SDS. The SNR at 550 nm was 5 dB, while it was 25 dB at 650 nm at the same SDS. Therefore, the optimal short channel uses the wavelength of 650 nm at a 2 mm SDS. For the long channel, the maximal dermis sensitivity for all wavelengths was between 4.5 and 8 mm SDS. As shown in Figure 4, the dermis sensitivity decreased with increasing wavelength. The optimal long channel uses a wavelength of 650 nm with an SDS between 4.5 and 6 mm. 

For darker skin (20% and 30% melanin concentration), the challenge for choosing the short channel was that the SNR was attenuated very quickly with increasing the SDS, specifically for the wavelengths of 450 and 550 nm, which had the highest epidermis sensitivity. Therefore, one must choose a very short SDS, of 1.5 mm or less, at 550 nm. For the long channel, one can choose 750 nm at a 4 to 5 mm SDS. However, for very dark skin (30% melanin concentration), one must assume a longer channel (950 or 1050 nm) to ensure a good SNR. Table 2 summarizes the selections of the wavelengths of the sources and the SDSs for the optimal NIR channels under different skin melanin concentrations.

## 4. Conclusions

In this work, we investigated the selection of the optimal dual-NIR channels for glucose measurements under different skin melanin concentrations, specifically for the diagnostic window of the NIR spectrum. The selection was based on the SNR and the sensitivity of both the epidermis and dermis layers considering different skin melanin concentrations. The detailed skin layer model that was adopted through MCS allowed us to take into consideration the differences between different skin layers, in terms of blood volume fraction, water volume fraction, melanin concentration in the epidermis layer, and optical skin proprieties. Since this work focused on the design of the dual-channel and the verifications of its parameters using MC simulation, future work should be experimentally conducted. Future work should also investigate the signal processing for this sensor and may include adopting an estimation model to filter the “noise” measured by the short channel. 

## Figures and Tables

**Table 1:** Values utilized in Equation (1) for the estimation of the absorption coefficients. The values were taken from [31].

Skin Layer	*V_blood_*	$V_{H_{2}O}$	Thickness (mm)
Stratum corneum	0	0.05	0.02 mm
Epidermis	0	0.2	0.25 mm
Papillary dermis	0.04	0.5	0.1 mm
Upper blood net dermis	0.3	0.6	0.08 mm
Reticular dermis	0.04	0.7	0.2 mm
Deep blood net dermis	0.1	0.7	0.3 mm
Subcutaneous tissue	0.05	0.7	2 mm

**Table 2:** Summary of suggested optimal NIR channels.

Melanin Concentration	Optimal for Short Channel		Optimal for Long Channel	
	Wavelength	SDS	Wavelength	SDS
2%	550 nm	2.5 mm	650 nm	4–6 mm
10%	650 nm	2 mm	650 nm	4–6 mm
20%	550 nm	1.5 mm	750 nm	4–5 mm
30%	550 nm	1.5 mm	950/1050 nm	4–5 mm

## Data Availability

The data presented in this study are openly available in FigShare at https://doi.org/10.6084/m9.figshare.21222452.
